# Multiple Arrhythmogenic Substrate for Tachycardia in a Patient with Frequent Palpitations

**Published:** 2005-01-01

**Authors:** Majid Haghjoo, Arash Arya, Mohammadreza Dehghani, Zahra Emkanjoo, Amirfarjam Fazelifar, MohammadAli Sadr-Ameli

**Affiliations:** Department of Pacemaker and Electrophysiology, Shahid Rajaie Cardiovascular Center, School of Medicine, Iran University of Medical Sciences, Tehran, Iran

**Keywords:** atrioventricular nodal reentrant tachycardia, atrioventricular reentrant tachycardia, atrial tachycardia, accessory pathway, catheter ablation

## Abstract

We report a 26-year-old woman with frequent episodes of palpitation and dizziness. Resting electrocardiography showed no evidence of ventricular preexcitation. During electrophysiologic study, a concealed right posteroseptal accessory pathway was detected and orthodromic atrioventricular reentrant tachycardia incorporating this pathway as a retrograde limb was reproducibly induced. After successful ablation of right posteroseptal accessory pathway, another tachycardia was induced using a concealed right posterolateral accessory pathway in tachycardia circuit. After loss of retrograde conduction of second accessory pathway with radiofrequency ablation, dual atrioventricular nodal physiology was detected and typical atrioventricular nodal reentrant tachycardia was repeatedly induced. Slow pathway ablation was done successfully. Finally sustained self-terminating atrial tachycardia was induced under isoproterenol infusion but no attempt was made for ablation. During 8-month follow-up, no recurrence of symptoms attributable to tachycardia was observed.

## Introduction

Several reports demonstrated the existence of double tachycardia such as atrioventricular reentrant tachycardia (AVRT) and atrioventricular nodal reentrant tachycardia (AVNRT) [1-3], or AVNRT and atrial tachycardia (AT)4, but coexistence of four different type of supraventricular tachycardia (SVT) in the same patient rarely reported. We report radiofrequency (RF) catheter ablation of two different AVRTs, and typical AVNRT in a patient with four different SVT.

## Case report

A 26-year-old woman, with no evidence of structural heart disease, was referred to our center for evaluation of palpitation and dizziness. Despite frequent occurrence of palpitation, she had no chance to capture any 12-lead electrocardiogram (ECG) during palpitation. The only evidence of arrhythmia in this patient was a sustained SVT with heart rate about 185 beats/min recorded in one of her 24-hour holter monitoring. Two antiarrhythmic agents (propranolol, verapamil) were tried in our patient with no success.

The baseline standard 12-lead ECG showed no any abnormality. The physical examination and transthoracic echocardiography were unremarkable.

**Figure 1:**
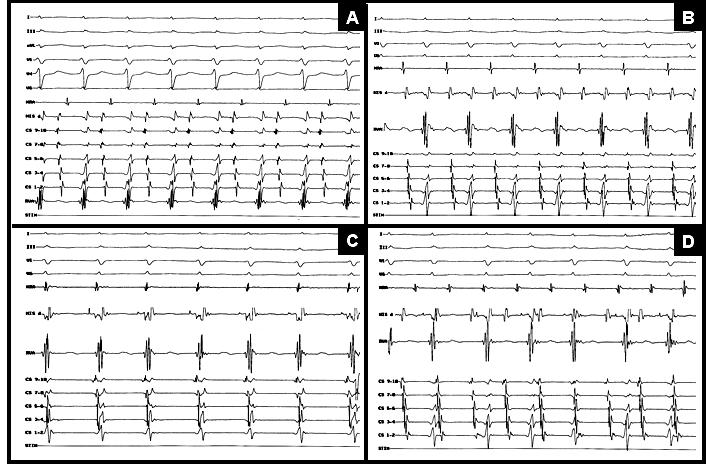
Four different types of SVTs induced during EPS. A, a narrow complex tachycardia (CL=320 ms) with earliest atrial activity in CS 9-10, compatible with AVRT using bypass tract as retrograde limb of tachycardia. B, another narrow complex tachycardia (CL=340 ms) induced after ablation of RPS-AP. Note that earliest atrial activity was recorded in HRA catheter, compatible with RFW-AP. C, typical AVNRT (CL=360 ms) induced after ablation of both right-sided APs. D, self-terminating AT induced after SP ablation. SVT= supraventricular tachycardia; EPS =electrophysiologic study; CL= cycle length; AVRT=atrioventricular reentrant tachycardia; AVNRT=atrioventricular nodal reentrant tachycardia; RPS-AP=right posteroseptal accessory pathway; HRA=high right atrium; RFW-AP=right free-wall accessory pathway; AT=atrial tachycardia; SP=slow pathway; HIS=His recording catheter; RVA=right ventricular apex.

After obtaining of written informed consent, electrophysiological study (EPS) was done in post absorptive, and non-sedated state. All antiarrhythmic drugs were interrupted for at least five half-lives before procedure. Three 6F diagnostic catheters (Daig, St. Jude Medical Inc., Minnetonka, MN, USA) were inserted via left femoral vein and placed in the high right atrium (HRA), His bundle position, and right ventricular apex (RVA), respectively. A 7F decapolar catheter with 2/2/2 mm electrode spacing (Marinr® CS; Medtronic, Inc., Minneapolis, MN, USA) positioned retrogradely via right femoral vein into the coronary sinus (CS) for coronary sinus mapping. During programmed electrical stimulation (PES) from RVA, nondecremental retrograde conduction was seen with earliest atrial activity on the proximal pole of CS catheter (located at the ostium of CS). No ventricular preexcitation was observed on atrial incremental pacing, compatible with a concealed right posteroseptal (RPS) accessory pathway. During atrial extrastimulation, a sustained narrow complex tachycardia (cycle length=320 ms) was induced reproducibly with earliest retrograde atrial activity in the proximal CS (Figure 1A). This tachycardia easily terminated by overdrive ventricular pacing and timed ventricular extrasystole during His refractoriness advanced atrial activity.

**Figure 2:**
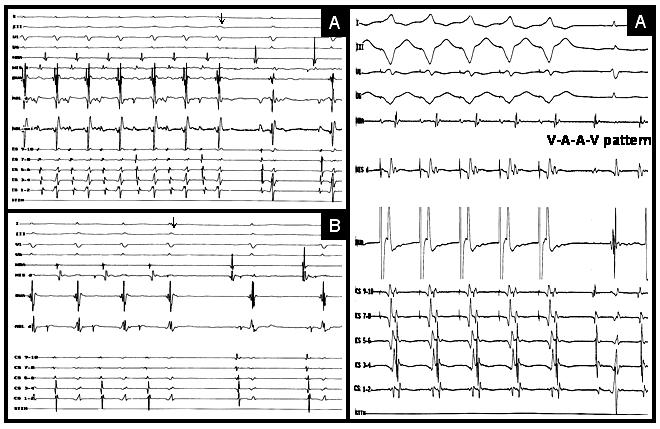
A, and B, termination of two forms of AVRTs using RPS-AP and RFW-AP following RF energy applications, respectively. C, ''VAAV'' pattern confirming atrial origin of tachycardia induced after SP ablation (for abbreviation, see the Figure 1).

Application of RF energy at right posteroseptal area resulted in loss of conduction over the accessory pathway (AP) (Figure 2AFigure 2A). Repeat ventricular PES revealed 1:1 vetriculoatrial conduction with earliest atrial activity on the HRA catheter, compatible with concealed right free wall (RFW) AP and atrial PES culminated in induction of a new narrow complex tachycardia (cycle length=340 ms) well-matched with orthodromic AVRT using RFW-AP (Figure 1B). Atrial advancement was also seen during the second AVRT (RFW). Mapping of tricuspid annulus localized the site of second AP on the posterolateral area. RF energy delivery at this area interrupted AP conduction (Figure 2B) and VA conduction shifted to the normal pathway. Repeat PES under isoproterenol infusion leads to induction of a new narrow complex tachycardia (cycle length=360 ms) compatible with typical AVNRT (Figure 1C). AVNRT became noninducible after successful slow pathway (SP) ablation. During postablation arrhythmia induction under isoproterenol infusion, a sustained, self-terminating atrial tachycardia was induced but no attempt was made for ablation (Figure 1D). Atrial origin of tachycardia was confirmed by V-A-A-V pattern after termination of ventricular pacing during tachycardia (Figure 2C). She left the EP laboratory in good condition without any complications.

During 8-month’s follow-up, she was free of symptoms with no antiarrhythmic drugs and no recurrence of tachycardia was seen.

## Discussion

The combination of AVNRT-AVRT [1-3] (including multiple bypass tracts), AVNRT-AT [4], and AVRT-AT [5] was reported. To the best of our knowledge, our case is the first patient reported with a combination of two AVRT, typical AVNRT, and AT.

As atrioventricular (AV) bypass tract is a congenital abnormality due to developmental defect in the AV rings, it is not surprising that multiple bypass tracts can be present in the same patient. Incidence of multiple bypass tracts ranges from 3.1-30% [6-8], and the most common combination has been RPS with RFW bypass tracts [6]. The incidence of multiple APs is higher in: 1) patients with antidromic AVRT; 2) patients in whom atrial fibrillation results in ventricular fibrillation and; 3) patients with Ebstein anomaly [9].

Dual AV nodal physiology is known to occur in 8-40% of patients with AP, leading to a variety of possible reentrant circuits [10,11]. In the study of Csanadi Z et al [12]., the most common arrhythmia in these patients were AVRT without AVNRT (75%), whereas 19% of patients had both AVRT and AVNRT, and 6% had only AVNRT. They also demonstrated that absence of clinical tachycardia during follow up in those with only dual AV nodal physiology with or without single echo cycle would argue against the routine ablation of the SP in these patients. At times, one tachycardia changes into another as depicted in an interesting case report [13].

In the study of 176 consecutive patients who underwent SP ablation for AVNRT, Sticherling et al [4]. demonstrated that 15% of patients with AVNRT, without prior clinically documented AT, were found to have inducible AT. Inducible AT was non-sustained in 74% of patients and induced only under isoproterenol infusion in the most of patients (74%). The sustained episodes terminated spontaneously after 30 seconds in  most of the patients. In this study, less than 10% of patients with inducible AT had recurrent AT in follow up period following SP ablation. This observation suggest that inducible AT, even when monomorphic and reproducibly inducible, often may be a non-specific finding that doesn't have clinical significance. Thus, therapy directed at the AT in patients who did not have documented prior AT should be deferred and limited to the occasional patients who later develop symptomatic AT.

Because of easy and reproducible induction of AVNRT after successful ablation of bypass tracts and absence of clinically documented tachycardia compatible with one of the specific types of SVT, we decided to ablate SP in this patient. Because the majority of patients with inducible AT without clinically sustained AT do not experience symptomatic atrial tachycardia during follow-up [4], ablation of AT was not attempted in this patient. The 8-month follow-up of our patient confirmed this decision.

## Conclusion

Our report demonstrated: 1) complexity of symptom origin in the patients with recurrent palpitations, 2) the importance of detailed electrophysiological assessment in the patients with AP before and after ablation, and 3) feasibility and efficacy of radiofrequency catheter ablation of multiple SVTs in single session.
